# Smooth Tubercle Bacilli: Neglected Opportunistic Tropical Pathogens

**DOI:** 10.3389/fpubh.2015.00283

**Published:** 2016-01-11

**Authors:** Djaltou Aboubaker Osman, Feriel Bouzid, Stéphane Canaan, Michel Drancourt

**Affiliations:** ^1^Aix-Marseille Université, URMITE, UMR CNRS 7278, IRD 198, INSERM 1095, Marseille, France; ^2^Centre d’Études et de Recherche de Djibouti (CERD), Institut de Recherche Médicinale (IRM), Djibouti, Republic of Djibouti; ^3^Enzymologie Interfaciale et Physiologie de la Lipolyse UMR7282, Centre National de la Recherche Scientifique (CNRS), Aix-Marseille Université, Marseille, France

**Keywords:** *Mycobacterium tuberculosis* complex, “*Mycobacterium canettii*”, smooth tubercle bacilli, Djibouti, Horn of Africa, amoebas, cellulases

## Abstract

Smooth tubercle bacilli (STB) including “*Mycobacterium canettii*” are members of the *Mycobacterium tuberculosis* complex (MTBC), which cause non-contagious tuberculosis in human. This group comprises <100 isolates characterized by smooth colonies and cordless organisms. Most STB isolates have been obtained from patients exposed to the Republic of Djibouti but seven isolates, including the three seminal ones obtained by Georges Canetti between 1968 and 1970, were recovered from patients in France, Madagascar, Sub-Sahara East Africa, and French Polynesia. STB form a genetically heterogeneous group of MTBC organisms with large 4.48 ± 0.05 Mb genomes, which may link *Mycobacterium kansasii* to MTBC organisms. Lack of inter-human transmission suggested a yet unknown environmental reservoir. Clinical data indicate a respiratory tract route of contamination and the digestive tract as an alternative route of contamination. Further epidemiological and clinical studies are warranted to elucidate areas of uncertainty regarding these unusual mycobacteria and the tuberculosis they cause.

## Introduction

In 2013, 9 million people developed tuberculosis (TB) and 1.5 million people infected with TB died (1). The vast majority of cases were caused by *Mycobacterium tuberculosis stricto sensu*, a cord-forming organism exhibiting rough colonies (2–4) while a few cordless isolates, referred as “smooth tubercle bacilli” (STB) were reported to form smooth colonies (5). The first three STB isolates made by Georges Canetti in 1968–1970 (6) were further named “*Mycobacterium canettii*” following the isolation of an additional STB isolate from a tuberculous lymph node in a Somali child (7). Then, a total of 93 STB have been isolated from patients exposed to tropical countries, mainly the Republic of Djibouti, which reports the highest prevalence and incidence of STB (5, 7–17). The reason for this geographical specificity is not really understood. Despite its rarity, STB deserve special attention due to their epidemiological, clinical, and microbiological characteristics, which are unique among the *M. tuberculosis* complex (MTBC).

## Particularities of the STB Infection

**Figure 1:**
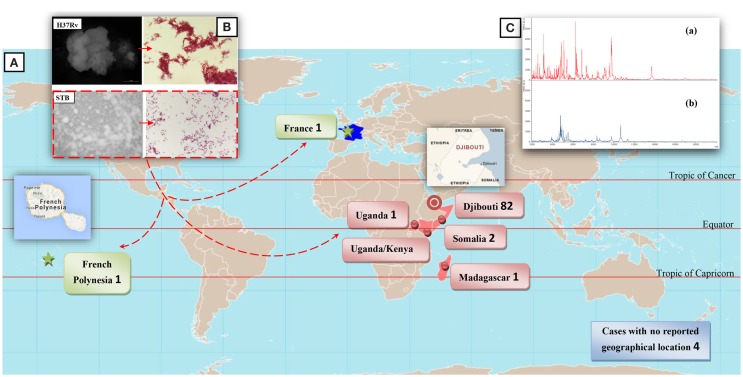
**(A)** Geographical sources for STB infection in 93 patients. **(B)** Aspects of STB and *M. tuberculosis* H37Rv colonies on 7H10 solid Middlebrook medium and Ziehl–Neelsen staining of mycobacteria. STB present smooth colonies and distribution of bacilli in singlets or aggregated small clumps instead of the cord-like aggregates usually seen with the rough H37Rv strains. **(C)** MALDI-TOF spectrum for “*M. canettii*” (a) and *M. tuberculosis* H37Rv (b).

**Figure 2:**
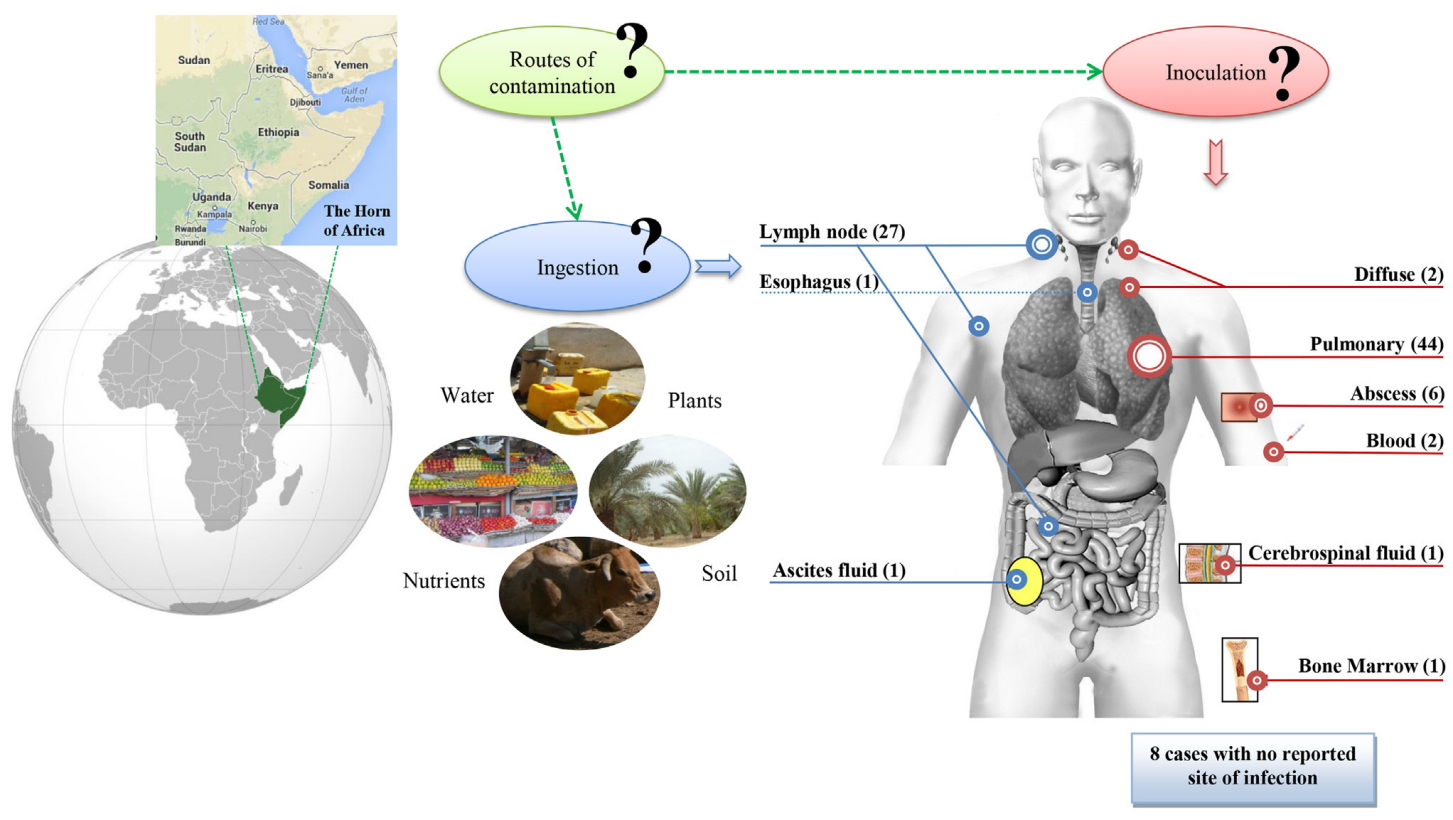
**STB tuberculosis anatomical sites of infection and potential environmental sources and routes of contamination**. Number of STB cases per site is indicated in brackets. Blue is for digestive tract, red for other anatomical sites. Question marks indicate hypothetical routes of contamination.

No environmental or animal STB isolates have been identified, contrary to that of *M. tuberculosis* (18). Indeed, the three seminal STB isolates were not reported by Canetti himself, but were rather identified through two indirect sources (6, 19). Accordingly, the precise history of these seminal isolates is poorly known, although it began prior to 1969, as deduced from a study on *M. tuberculosis* var. *hominis*, *Canetti* strain mycolic acids submitted for publication in 1968 (20). This first isolate was obtained from a 20-year-old French farmer suffering from pulmonary TB although he had apparently never left France (6, 19). Canetti obtained a second isolate from a 54-year-old farmer also suffering from pulmonary TB in Madagascar, then a third isolate from a man suffering tuberculous adenitis in Papeete, Tahiti (6, 19). Surprisingly, the first ever reported STB isolates were therefore from three patients with no reported contact with the Horn of Africa, where the vast majority of cases had been reported. In 1997, a fourth STB isolate (So93 strain) was reported as “*M. canettii*” (7). The more general term “STB” used here was quoted in a report on *M. tuberculosis* smooth variants in Djibouti (5). Since 1997, a survey of the literature found a total of 93 STB isolates, mainly obtained from patients exposed to tropical countries (Table S1 in Supplementary Material). Indeed, 82/93 (88%) isolates were obtained from patients exposed to the Republic of Djibouti, 2/93 (2%) from patients exposed to Uganda including one also exposed to Kenya, 2/93 (2%) from patients exposed to Somalia, 3 other patients exposed to France, French Polynesia, and Madagascar, and 4 cases with unknown geographical exposure (Figure 1A). With the notable exception of the three seminal isolates, all isolates were obtained between the 23° 26′ 16″ N and 23° 26′ 16″ S parallels in tropical countries with a coastline (Figure 1A). Following the description of the first cases in 1969 and 1970, few cases were reported between 1991 and 1997, although 29/93 cases were described from 1998 to 2000. A second peak in case reporting was observed between 2002 and 2003, with 17 cases being described, and a third peak took place 8 years later with 10 new cases (Figure S1 in Supplementary Material). It should be noted that the number of published cases significantly correlates to the number of STB papers published over the same time period (*P* = 1.208e − 13, Pearson’s correlation), suggesting a positive bias in reporting cases (Figure S1 in Supplementary Material). Interest in STB isolates gained ground around the 2000s, suggesting that efforts were concentrated where the main strains were collected, mainly in the Horn of Africa. Furthermore, the unusual macroscopic phenotype of the STB strains may delay their diagnosis and may even result in them being underreported. Clinical data available for 85/93 patients (5, 7, 8, 10, 11, 13–17) indicate 44/85 (52%) had the pulmonary form and 41/85 (48%) had the extra-pulmonary form, including lymph node involvement in 32% of cases (Figure 2). In Djibouti, no significant difference was found in the prevalence of the pulmonary form between STB [17/30 (56.6%)] and *M. tuberculosis stricto sensu* [2,188/3,772; (58%), *P* = 0, 88 > 0, 1, *X*^2^ test] (*Plan National de Lutte Anti Tuberculeuse*, 1997). However, the prevalence of enlarged lymph nodes in STB (12/30; 40%) was significantly higher than in *M. tuberculosis stricto sensu* (717/3,772; 19%) (*P* = 0.038, *X*^2^ test). In Djibouti, a recent epidemiological investigation found that all STB lymph nodes were diagnosed in children and that all STB children had lymph nodes which were infected (8). Indeed, the So93 strain was also obtained from lymphadenitis in a 2-year-old Somali child (7). Of note, the age of children with STB lymph nodes in the Horn of Africa shows a bimodal distribution with 7/14 children ≤4 years. This is the median age reported for *Mycobacterium avium hominissuis* lymph nodes (21). This observation suggests that young children are infected by suction of contaminated fomites. These clinical observations suggest an oropharyngeal portal of entry for STB. Moreover, reports of STB-infected mesenteric lymph nodes (15) as well as one case of STB ascites (19) suggest a digestive tract route of infection in addition to the respiratory tract route. The establishment of an animal model using an oral route for STB infection could evaluate the possibility of STB infection through digestive tract route. Interestingly, in contrast to classical TB infection, there is no evidence of human-to-human transmission of STB infection, suggesting the existence of an as yet unknown environmental reservoir (5). Accordingly, “*M. canettii*” (CIPT140010059) was shown to survive in experimentally infected soil for a minimum of 12 months (22). Taken together, these observations suggest that soil may be a direct or indirect source of STB through drinking water and food, entering and replicating at the oropharyngeal portal of entry and spreading into the respiratory and digestive tracts (Figure 2).

## Particularities of the STB Organisms

The generation time of STB is two to three times shorter than that of *M. tuberculosis* strains in both liquid media and solid media at 30°C and 37°C (3 and 8 days for STB and *M. tuberculosis* H37Rv, respectively, at 37°C as measured by BACTEC 460 System in numerical growth units), a feature also of *Mycobacterium microti* (9). By definition, STB present smooth colonies, which are white to pale beige and glossy (Figure 1B) (5) correlating with the presence of a large amount of triglycosyl glycolipids (7, 23, 24). Through electron microscopy scanning, colonies were observed to vary from small, singular, flat and cone-shaped to larger compound colonies formed by a homogeneous distribution of bacilli in singlets or aggregated in small clumps instead of the cord-like aggregates usually seen with rough MTBC strains (7) (Figure 1B). Specific biochemical traits, including antibiotic susceptibility patterns, are reported in Table S2 in Supplementary Material. Matrix-Assisted Laser Desorption Ionization-Time-of-Flight Mass Spectrometry (MALDI-TOF-MS) fingerprinting (25) yields a distinctive peptide spectrum for “*M. canettii*” (Figure 1C). Five available whole STB genomes indicate a 4.4202–4.52595 Mb chromosome larger than that of the other MTBC members. This difference is reflected by a set of 890 predicted coding sequences (~20%) present in STB and absent in the other members of the MTBC (17). However, 14/890 (1.6%) genes only are common to all five genome-sequenced STB strains (Table S3 in Supplementary Material) (17). The rest of these genes are variably distributed between the different STB strains (17). Whereas the evolutionary of *M. tuberculosis* is mainly characterized by a genome size reduction linked to gene loss and host adaptation, STB still carry traces of interactions with donor organisms suggesting that STB are environmental organisms, which retain a broad spectrum adaptative capability (17). No phages have been observed, but a controversial 55-kb prophage was identified in STB-I (17, 26), nine spacers matching the *Mycobacterium marinum* strain M prophage, and two spacers matching the Thibault or Redi *Mycobacterium* phages. Three additional prophages, phiBN42_1, phiBN44_1, phiMCAN_1, have been described respectively as “*M. canettii*” CIPT 140070010, “*M. canettii*” CIPT 140060008 and “*M. canettii*” CIPT 140010059 (26). These prophages may play a major role in the evolution of STB, as previously reported for *M*. abscessus (27). Further study found that some STB isolates lacked the insertion element *IS*1081, while a new *ISMycA1* (GenBank accession number AJ619854) was discovered in the “*M. canettii*” CIPT140010059 genome (12). IS*MycA1* encodes a transposase which, surprisingly, shares 48% amino acid sequence identity with IS-encoded transposases of the *Mycobacterium ulcerans* plasmid (28). IS*MycA1* is a distinctive characteristic of STB in comparison with the other MTBC members (12). Indeed, the original “*M. canettii”* strain (CIPT 140010059) and So93 are indistinguishable from the other MTBC members as a result of sequencing of 16S rRNA and housekeeping genes (*rpo*B, *kat*G, *rps*L, and *gyr*A) (7). Nevertheless, further analysis of six housekeeping genes yielded 14 (A-N) STB clonal groups (12, 17). The multiple locus variable number of tandem repeats analysis (MLVA) (10, 11) highlighted that ETR-A (allele 10), ETR-C (alleles 6 and 10), MIRU-02 (allele 3), MIRU-40 (allele 8), and Mtub29 (allele 5) were unique to STB strains (10). Compared to *M*. *tuberculosis* H37Rv, investigations showed the presence of an intact region of deletion RD9 and the *M. tuberculosis* specific deletion (TbD1) (11, 29, 30). Indeed, TbD1 region is present in 59 STB strains tested including the seminal isolate “*M. canettii”* CIPT140010059; along with 11 West African *M. africanum* isolates and 20 *Mycobacterium bovis* including two BCG strains. At the opposite, 40 of 46 tested *M. tuberculosis* strains were TbD1 deleted comprising representatives from major tuberculosis epidemics such as the Beijing, Haarlem, African *M. tuberculosis* clusters, and the seminal isolates made by Robert Kock in 1882 (11, 29). The region TbD1 contains two genes encoding two uncharacterized membrane proteins, *mmpS6* gene (Mycobacterium Membrane Protein Small) and *mmpL6* gene (Mycobacterium Membrane Protein Large). In *M. tuberculosis* H37Rv, *mmpS6* is absent and *mmpL6* is truncated.

Genomic analysis revealed that the precorrin gene *cob*F, preserved in many environmental mycobacteria, including *Mycobacterium kansasii* (31), is also present in all STB but is absent in all other MTBC members (8, 17). In STB, repetitive sequences of the PE-PGRS families are highly diverse; in particular, PE_PGRS62 is polymorphic and positively selected in STB, while it is highly preserved in MTBC (31). Indeed, STB strains show unprecedented high genetic heterogeneity with traces of intra-species horizontal gene transfer (HGT) compared to the worldwide population of MTBC strains, which represent one of the most extreme examples of a genetically homogeneous group (8, 12, 17). Recently, distributive conjugal transfer was found to be a predominant mechanism for lateral gene transfer among STB, supporting the high heterogeneity observed in this group (32, 33). This mechanism provides an incomparable means for generating rapidly remarkable genetic diversity in a single step, which makes each strain uniquely different from the others (32). Thus a few STB isolates from a geographically restricted region, the Horn of Africa, show a larger genetic diversity than the world-wide population of MTBC strains. These observations led to a new evolutionary scenario for the emergence of pathogenic *M. tuberculosis* from an environmental organism, such as *M. kansasii*, through transitional “smooth” tubercle bacilli (34–36).

## STB Infection Models

Only amoebas have been used as a cell model for “*M. canettii*” infection (37). In this model, 89% of “*M. canettii*” organisms, which were co-cultured with free-living *Acanthamoeba polyphaga* ameba were ingested by trophozoites, a ratio which is significantly higher than for *M. tuberculosis*, *M. bovis*, and *M. avium* (37). This difference correlates with a 2.56 μm larger size for “*M. canettii*” and smoothness reflecting the specific presence of glycolipid containing triglycosyl. In a *M. marinum*–*Acanthamoeba* coculture model, it was shown that lipooligosaccharide modulates the phagocytosis of mycobacteria in *Acanthamoeba* (38). In contrast to *M. tuberculosis* and *M. bovis*, “*M. canettii*” survives into cytoplasmic vacuoles and escapes from encystment (37). This specific behavior could be related to the activation of cellulases Cel6, Cel12 and CBD2 to lyse the cellulose cell wall of the amoebal exocyst (39, 40). In the absence of any known reservoir (5), further studies presenting animal models with contradictory results may not be relevant to natural human infection. A first model of guinea pigs, which were inoculated subcutaneously and intramuscularly with 1 mL 10^3^ or 10^5^ colony-forming units (CFU) of So93 or *M. tuberculosis* H37Rv did not show signs of clinical disease for 8 weeks (7). However, necropsy found overwhelming disseminated tuberculous lesions and severe loss of body fat deposits in guinea pigs inoculated with So93, in contrast to animals inoculated with *M. tuberculosis* H37Rv. In all animals, it has been found that the liver, spleen as well as the lungs were infected. Virulence, measured by microscopic and bacteriological examination and average root index of virulence calculation, was lower for *M. tuberculosis* H37Rv than for So93 (7). In a further study, BALB/c mice were infected intratracheally by 2 × 10^5^ viable cells of “*M. canettii*” (strains CIPT 140010059 and So93) or *M. tuberculosis* H37Rv (41). Two and 3 weeks after infection, “*M. canettii*” induced larger perivascular infiltrates and significantly smaller areas of granuloma in the lung than *M. tuberculosis* H37Rv. Also, “*M. canettii*” CIPT 140010059 induced sustained TNF-α and iNOS expression in lungs combined with delayed and moderate IFN-γ expression. Four-week post-infection, “*M. canettii*” strains yielded almost 100% survivals significantly higher than 40–50% survivals in *M. tuberculosis*-infected animals. In addition, lung replication of “*M. canettii*” strains was significantly lower than that of *M. tuberculosis* H37Rv at all time points. At the final time point, pneumonic areas induced by the “*M. canettii*” CIPT 140010059 were significantly smaller than those produced by *M. tuberculosis* H37Rv (41). In a further model, BALB/c mice were infected intratracheally with 2.5 × 10^5^ viable cells of “*M. canettii*” CIPT 140010059 or ten major genotypes of *M. tuberculosis* (H37Rv, Africa, Amesterdam, Beijing, Erdman, Haarlem, IS-in-Ori, Less-trans, Somalia, Zerocopy) (42). “*M. canettii*” and *M. tuberculosis* H37Rv did not induce lung pathology for 3 weeks, and “*M. canettii*” caused limited pneumonia with mild peribronchiolitis, perivasculitis and alveolitis in the absence of granuloma formation at day 56 post-infection; at day 120 post-infection, “*M. canettii*” and *M. tuberculosis* H37Rv yielded a similar 10% death rate (42). Additional animal models were conducted by infecting BALB/c and C57BL/6 mice with 10^3^ CFUs of STB-D, STB-L, STB-K, or STB-J, *M. tuberculosis* TbD1 positive or *M. tuberculosis* TbD1 negative by intranasal aerosol (17). The STB strains effectively multiplied in the lungs and disseminated to the spleen 3 weeks after inoculation, but consistently persisted for less time during the chronic infection phase (30 weeks), compared to both *M. tuberculosis* strains. Furthermore, 128 days after inoculation, histopathological analyses revealed less severe lung lesions and inflammation in STB-infected mice than in *M. tuberculosis* infected mice (17). The lower virulence and persistence of STB strains correlated to differences in both innate and adaptive immune responses (17). In infected SCID mice, recruitment of activate innate cells was observed in the lung parenchyma 3-week post-infection with STB to a lower extent compared to *M. tuberculosis* infection. In addition, 13-week post-infection lung recruitment of activated CD4^+^ and CD8^+^ lymphocytes was quantitatively lower in STB-infected mice compared to *M. tuberculosis*-infected mice (17).

## Conclusion

With <100 reported cases, STB infection remains a neglected infectious disease in tropical countries in East Africa. Indeed, their unique morphological features, which are unusual among the MTBC, with smooth, shiny luxuriant, and rapidly growing colonies, may lower their presumptive identification as MTBC members. Their cordless appearance observed after Ziehl–Neelsen staining further complicates first-line identification in endemic countries. The reservoirs and mode of transmission remain unknown but comparing clinical data with scarce experimental data suggests contaminated drinking water and food as potential sources, with local replication in the oropharynx and cervical lymph nodes and further dissemination in the respiratory and digestive tracts. In terms of this hypothesis, looking for STB in the stools of patients would be of interest, as it has been observed in patients with *M. tuberculosis* pulmonary tuberculosis (43, 44). Likewise, genetic and genomic data including large genome size and the abundance of phage sequences, suggest that STB form a heterogeneous group of tuberculosis organisms with intermediate features in between mammal-adapted *M. tuberculosis* organisms and environmental organisms such as *M. kansasii* (36). By means of conclusion, the data reviewed here could form the foundation of efforts toward elucidating the reservoirs and sources of STB, along with the development of laboratory tests aimed at a point-of-care diagnosis of STB infection (45).

## Conflict of Interest Statement

The authors declare that the research was conducted in the absence of any commercial or financial relationships that could be construed as a potential conflict of interest. The reviewer Dr Gyanu Lamichhane and handling Editor Dr Ying Zhang declare their shared affiliation, and the handling Editor states that the process nevertheless met the standards of a fair and objective review.

## References

[1] WHO (World Health Organization). Global Tuberculosis Report. (2014). Available from: http://apps.who.int/iris/bitstream/10665/137094/1/9789241564809_eng.pdf

[2] Julian E Roldan M Sanchez-Chardi A Astola O Agusti G Luquin M. Microscopic cords, a virulence-related characteristic of *Mycobacterium tuberculosis*, are also present in nonpathogenic mycobacteria. J Bacteriol (2010) 192:1751–60. 10.1128/JB.01485-09 20097851 PMC2838037

[3] Middlebrook G Dubos RJ Pierce C. Virulence and morphological characteristics of mammalian tubercle bacilli. J Exp Med (1947) 86:175–84. 10.1084/jem.86.2.175 19871665 PMC2135722

[4] Runyon EH Identification of mycobacterial pathogens utilizing colony characteristics. Am J Clin Pathol (1970) 54:578–86. 5471228 10.1093/ajcp/54.4.578

[5] Koeck JL Fabre M Simon F Daffe M Garnotel E Matan AB Clinical characteristics of the smooth tubercle bacilli ‘*Mycobacterium canettii*’ infection suggest the existence of an environmental reservoir. Clin Microbiol Infect (2011) 17:1013–9. 10.1111/j.1469-0691.2010.03347.x 20831613

[6] Goh KS Legrand E Sola C Rastogi N. Rapid differentiation of “*Mycobacterium canettii” f*rom other *Mycobacterium tuberculosis* complex organisms by PCR-restriction analysis of the hsp65 gene. J Clin Microbiol (2001) 39:3705–8. 10.1128/JCM.39.10.3705-3708.2001 11574597 PMC88413

[7] van Soolingen D Hoogenboezem T de Haas PE Hermans PW Koedam MA Teppema KS A novel pathogenic taxon of the *Mycobacterium tuberculosis* complex, Canetti: characterization of an exceptional isolate from Africa. Int J Syst Bacteriol (1997) 47:1236–45. 10.1099/00207713-47-4-1236 9336935

[8] Blouin Y Cazajous G Dehan C Soler C Vong R Hassan MO Progenitor “*Mycobacterium canettii”* clone responsible for lymph node tuberculosis epidemic, Djibouti. Emerg Infect Dis (2014) 20:21–8. 10.3201/eid2001.130652 24520560 PMC3884719

[9] Comas I Chakravartti J Small PM Galagan J Niemann S Kremer K Human T cell epitopes of *Mycobacterium tuberculosis* are evolutionarily hyperconserved. Nat Genet (2010) 42:498–503. 10.1038/ng.590 20495566 PMC2883744

[10] Fabre M Koeck JL Le Fleche P Simon F Herve V Vergnaud G High genetic diversity revealed by variable-number tandem repeat genotyping and analysis of hsp65 gene polymorphism in a large collection of “*Mycobacterium canettii”* strains indicates that the *M. tuberculosis* complex is a recently emerged clone of “*M. canettii”*. J Clin Microbiol (2004) 42:3248–55. 10.1128/JCM.42.7.3248-3255.2004 15243089 PMC446256

[11] Fabre M Hauck Y Soler C Koeck JL van Ingen J van Soolingen D Molecular characteristics of “*Mycobacterium canettii”* the smooth *Mycobacterium tuberculosis* bacilli. Infect Genet Evol (2010) 10:1165–73. 10.1016/j.meegid.2010.07.016 20692377

[12] Gutierrez MC Brisse S Brosch R Fabre M Omais B Marmiesse M Ancient origin and gene mosaicism of the progenitor of *Mycobacterium tuberculosis*. PLoS Pathog (2005) 1:e5. 10.1371/journal.ppat.0010005 16201017 PMC1238740

[13] Hugard L Dubrous P Massoure PL Renoux E Desemerie F [*Mycobacterium canettii* in a tuberculous patient having stayed in Africa]. Med Mal Infect (2004) 34(3):142–3. 10.1016/j.medmal.2003.12.005 15617357

[14] Miltgen J Morillon M Koeck JL Varnerot A Briant JF Nguyen G Two cases of pulmonary tuberculosis caused by *Mycobacterium tuberculosis* subsp canetti. Emerg Infect Dis (2002) 8:1350–2. 10.3201/eid0811.020017 12453369 PMC2738533

[15] Pfyffer GE Auckenthaler R van Embden JD van Soolingen D. *Mycobacterium canettii*, the smooth variant of M. tuberculosis, isolated from a Swiss patient exposed in Africa. Emerg Infect Dis (1998) 4:631–4. 10.3201/eid0404.980414 9866740 PMC2640258

[16] Somoskovi A Dormandy J Mayrer AR Carter M Hooper N Salfinger M. “*Mycobacterium canettii”* isolated from a human immunodeficiency virus-positive patient: first case recognized in the United States. J Clin Microbiol (2009) 47:255–7. 10.1128/JCM.01268-08 19020064 PMC2620870

[17] Supply P Marceau M Mangenot S Roche D Rouanet C Khanna V Genomic analysis of smooth tubercle bacilli provides insights into ancestry and pathoadaptation of *Mycobacterium tuberculosis*. Nat Genet (2013) 45:172–9. 10.1038/ng.2517 23291586 PMC3856870

[18] Ghodbane R Drancourt M Non-human sources of *Mycobacterium tuberculosis*. Tuberculosis (2013) 93:589–95. 10.1016/j.tube.2013.09.005 24119770

[19] Bruno D Tuberculoses à *Mycobacterium Canetti* Epidémiologie, clinique, microbiologie et phylogénie. Medical Thesis, Bordeaux, France, 2006.

[20] Asselineau C Montrozier H Prome JC [Structure of alpha-mycolic acids isolated from the Canetti strain of *Mycobacterium tuberculosis*]. Bull Soc Chim Fr (1969) 2:592–6. 4979218

[21] Despierres L Cohen-Bacrie S Richet H Drancourt M Diversity of *Mycobacterium avium* subsp. *hominissuis* mycobacteria causing lymphadenitis, France. Eur J Clin Microbiol Infect Dis (2012) 31:1373–9. 10.1007/s10096-011-1452-2 22042560

[22] Ghodbane R Mba Medie F Lepidi H Nappez C Drancourt M. Long-term survival of tuberculosis complex mycobacteria in soil. Microbiology (2014) 160:496–501. 10.1099/mic.0.073379-0 24425768

[23] Daffe M McNeil M Brennan PJ. Novel type-specific lipooligosaccharides from *Mycobacterium tuberculosis*. Biochemistry (1991) 30:378–88. 10.1021/bi00216a011 1899023

[24] Daffe M Lacave C Laneelle MA Laneelle G. Structure of the major triglycosyl phenol-phthiocerol of *Mycobacterium tuberculosis* (strain Canetti). Eur J Biochem (1987) 167:155–60. 10.1111/j.1432-1033.1987.tb13317.x 3113946

[25] El Khechine A Couderc C Flaudrops C Raoult D Drancourt M. Matrix-assisted laser desorption/ionization time-of-flight mass spectrometry identification of mycobacteria in routine clinical practice. PLoS One (2011) 6:e24720. 10.1371/journal.pone.0024720 21935444 PMC3172293

[26] Fan X Xie L Li W Xie J. Prophage-like elements present in Mycobacterium genomes. BMC Genomics (2014) 15:243. 10.1186/1471-2164-15-243 24673856 PMC3986857

[27] Sassi M Gouret P Chabrol O Pontarotti P Drancourt M. Mycobacteriophage-drived diversification of *Mycobacterium abscessus*. Biol Direct (2014) 9:19. 10.1186/1745-6150-9-19 25224692 PMC4172396

[28] Stinear TP Pryor MJ Porter JL Cole ST. Functional analysis and annotation of the virulence plasmid pMUM001 from *Mycobacterium ulcerans*. Microbiology (2005) 151:683–92. 10.1099/mic.0.27674-0 15758215

[29] Brosch R Gordon SV Marmiesse M Brodin P Buchrieser C Eiglmeier K A new evolutionary scenario for the *Mycobacterium tuberculosis* complex. Proc Natl Acad Sci USA (2002) 99:3684–9. 10.1073/pnas.052548299 11891304 PMC122584

[30] Marmiesse M Brodin P Buchrieser C Gutierrez C Simoes N Vincent V Macro-array and bioinformatic analyses reveal mycobacterial ‘core’ genes, variation in the ESAT-6 gene family and new phylogenetic markers for the *Mycobacterium tuberculosis* complex. Microbiology (2004) 150:483–96. 10.1099/mic.0.26662-0 14766927

[31] Namouchi A Karboul A Fabre M Gutierrez MC Mardassi H. Evolution of smooth tubercle Bacilli PE and PE_PGRS genes: evidence for a prominent role of recombination and imprint of positive selection. PLoS One (2013) 8:e64718. 10.1371/journal.pone.0064718 23705005 PMC3660525

[32] Gray TA Krywy JA Harold J Palumbo MJ Derbyshire KM. Distributive conjugal transfer in mycobacteria generates progeny with meiotic-like genome-wide mosaicism, allowing mapping of a mating identity locus. PLoS Biol (2013) 11:e1001602. 10.1371/journal.pbio.1001602 23874149 PMC3706393

[33] Mortimer TD Pepperell CS. Genomic signatures of distributive conjugal transfer among mycobacteria. Genome Biol Evol (2014) 6(9):2489–500. 10.1093/gbe/evu175 25173757 PMC4202316

[34] Minnikin DE Lee OY Wu HH Besra GS Bhatt A Nataraj V Ancient mycobacterial lipids: key reference biomarkers in charting the evolution of tuberculosis. Tuberculosis (2015) 95(Suppl 1):S133–9. 10.1016/j.tube.2015.02.009 25736170

[35] Wang J Behr MA. Building a better bacillus: the emergence of *Mycobacterium tuberculosis*. Front Microbiol (2014) 5:139. 10.3389/fmicb.2014.00139 24765091 PMC3982062

[36] Wang J McIntosh F Radomski N Dewar K Simeone R Enninga J Insights on the emergence of *Mycobacterium tuberculosis* from the analysis of *Mycobacterium kansasii*. Genome Biol Evol (2015) 7:856–70. 10.1093/gbe/evv035 25716827 PMC5322544

[37] Mba Medie F Ben Salah I Henrissat B Raoult D Drancourt M. *Mycobacterium tuberculosis* complex mycobacteria as amoeba-resistant organisms. PLoS One (2011) 6:e20499. 10.1371/journal.pone.0020499 21673985 PMC3108610

[38] Alibaud L Pawelczyk J Gannoun-Zaki L Singh VK Rombouts Y Drancourt M Increased phagocytosis of *Mycobacterium marinum* mutants defective in lipooligosaccharide production: a structure-activity relationship study. J Biol Chem (2014) 289:215–28. 10.1074/jbc.M113.525550 24235141 PMC3879545

[39] Mba Medie F Ben Salah I Drancourt M Henrissat B. Paradoxical conservation of a set of three cellulose-targeting genes in *Mycobacterium tuberculosis* complex organisms. Microbiology (2010) 156:1468–75. 10.1099/mic.0.037812-0 20150238

[40] Mba Medie F Vincentelli R Drancourt M Henrissat B *Mycobacterium tuberculosis* Rv1090 and Rv1987 encode functional beta-glucan-targeting proteins. Protein Expr Purif (2011) 75:172–6. 10.1016/j.pep.2010.08.015 20826214

[41] Lopez B Aguilar D Orozco H Burger M Espitia C Ritacco V A marked difference in pathogenesis and immune response induced by different *Mycobacterium tuberculosis* genotypes. Clin Exp Immunol (2003) 133:30–7. 10.1046/j.1365-2249.2003.02171.x 12823275 PMC1808750

[42] Dormans J Burger M Aguilar D Hernandez-Pando R Kremer K Roholl P Correlation of virulence, lung pathology, bacterial load and delayed type hypersensitivity responses after infection with different *Mycobacterium tuberculosis* genotypes in a BALB/c mouse model. Clin Exp Immunol (2004) 137:460–8. 10.1111/j.1365-2249.2004.02551.x 15320894 PMC1809137

[43] Bonnave PE Raoult D Drancourt M. Gastric aspiration is not necessary for the diagnosis of pulmonary tuberculosis. Eur J Clin Microbiol Infect Dis (2013) 32:569–71. 10.1007/s10096-012-1776-6 23143083

[44] El Khechine A Henry M Raoult D Drancourt M. Detection of *Mycobacterium tuberculosis* complex organisms in the stools of patients with pulmonary tuberculosis. Microbiology (2009) 155:2384–9. 10.1099/mic.0.026484-0 19389783

[45] Cohen-Bacrie S Ninove L Nougairede A Charrel R Richet H Minodier P Revolutionizing clinical microbiology laboratory organization in hospitals with in situ point-of-care. PLoS One (2011) 6:e22403. 10.1371/journal.pone.0022403 21811599 PMC3139639

[46] Wanger A Mills K. Testing of *Mycobacterium tuberculosis* susceptibility to ethambutol, isoniazid, rifampin, and streptomycin by using Etest. J Clin Microbiol (1996) 34:1672–6. 8784567 10.1128/jcm.34.7.1672-1676.1996 PMC229092

[47] Feuerriegel S Köser CU Niemann S *Mycobacterium canettii* is intrinsically resistant to both pyrazinamide and pyrazinoic acid. J Antimicrob Chemother (2003) 68:1439–50. 10.1093/jac/dkt042 23447141

