# Supplementary material for: Smooth Tubercle Bacilli: Neglected Opportunistic Tropical Pathogens
Source: Front Public Health. 2016 Jan 11;3:283. doi: 10.3389/fpubh.2015.00283 (PMC4707939; doi:10.3389/fpubh.2015.00283)
Supplement: Supplementary file 2 [file table_2.docx]

**Supplementary Table 2: Classification of STB in comparison with *M. tuberculosis* H37Rv.**

| Characteristics | STB | *M. tuberculosis* H37Rv | References |
| --- | --- | --- | --- |
| Morphological | | | |
| Colony appearance | Eugonic smooth | Eugonic rough | *(*[*5*](#_ENREF_5)*)* |
| Depth of growth | Aerophilic | Micro-aerophilic | *(*[*22*](#_ENREF_22)*)* |
| Doubling time in liquid Tween Albumin medium | 17 hours | 25 hours | *(*[*7*](#_ENREF_7)*)* |
| Generation time   - LJ medium - BACTEC 460 System | 22 (16–25 days)  3 days | 23 (18–25 days)  8 days | *(*[*5*](#_ENREF_5)*) (*[*17*](#_ENREF_17)*)* |
| Growth on minimum solid media (Trypticase-soy agar) | Positive | Negative | *(*[*5*](#_ENREF_5)*)* |
| Biochemical | | | |
| Nitrate reductase | Present | Present | *(*[*5*](#_ENREF_5)*)* |
| Niacin production | Absent | Present | *(*[*5*](#_ENREF_5)*)* |
| Drug susceptibility | | | |
| Pyrazinamide (PZA)  100 mg/L | Resistant | Sensitive | *(47)*  *(*[*15*](#_ENREF_15)*)*  *(*[*16*](#_ENREF_16)*)*  *(*[*46*](#_ENREF_46)*)* |
| Thiophen-2-carboxylic acid hydrazine (TCH)  2 mg/L | Resistant | Resistant |  |
| Streptomycin (SM) | 2 – 10 µg/mL | 0.25–1.0 µg/mL |  |
| Isoniazid (INH) | 0.2–1 µg/mL | 0.016–0.06 µg/mL |  |
| Rifampin (RIF) | 0.2 µg/mL | 0.06–0.25 µg/mL |  |
| Ethambutol (EMB) | 2.5 – 7.5 µg/mL | 0.06–0.25 µg/mL |  |
| Molecular | | | |
| Genome size (Mb) | 4.29797 – 4.52595 | 4.4115 | *(*[*17*](#_ENREF_17)*)* |
| GC% | 65.40 – 65.60 | 65.50 | *(*[*17*](#_ENREF_17)*)* |
| TbD1 region | Present | Absent | *(*[*29*](#_ENREF_29)*)* |
