# Supplementary figures and images for: Smooth Tubercle Bacilli: Neglected Opportunistic Tropical Pathogens
Source: Front Public Health. 2016 Jan 11;3:283. doi: 10.3389/fpubh.2015.00283 (PMC4707939; doi:10.3389/fpubh.2015.00283)

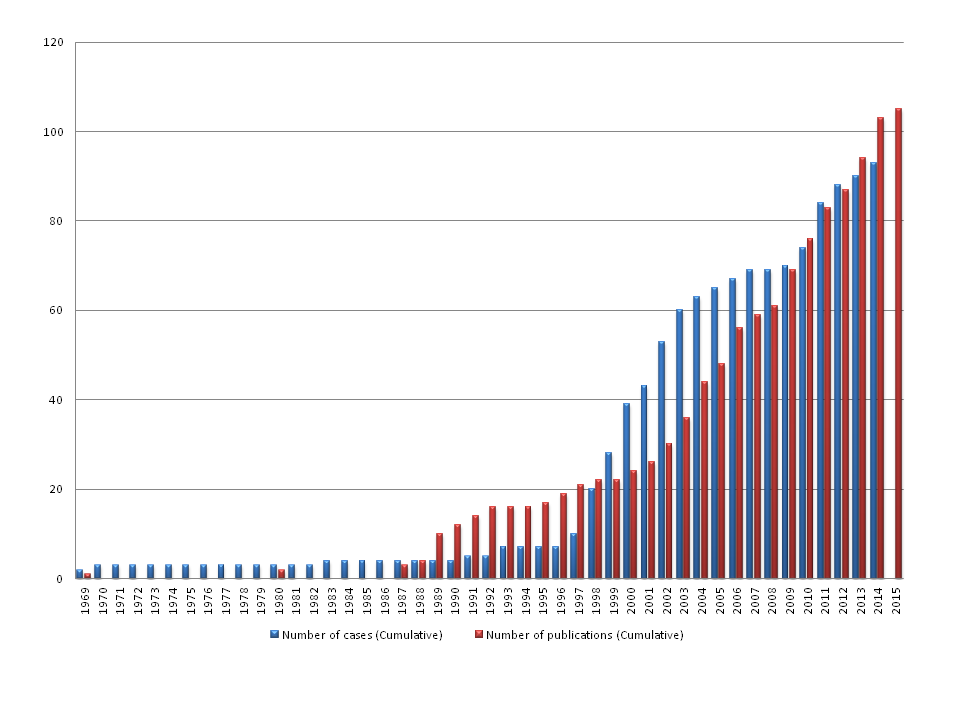

Supplement: Supplementary file 4 [file image_1.tif]
